# Prevalence, distribution and correlates of tobacco smoking and chewing in Nepal: a secondary data analysis of Nepal Demographic and Health Survey-2006

**DOI:** 10.1186/1747-597X-6-33

**Published:** 2011-12-20

**Authors:** Chandrashekhar T Sreeramareddy, N Ramakrishnareddy, HN Harsha Kumar, Brijesh Sathian, John T Arokiasamy

**Affiliations:** 1 Department of Clinical Sciences, Faculty of Medical and Health Science, University Tunku Abdul Rahman, Bandar Sungai Long, Kajang, Selangor, Malaysia; 2 Bangalore Medical College and Research Institute, Fort, Bangalore, India; 3 Kasturba Medical College, Manipal University, Mangalore, India; 4 Manipal College of Medical Sciences, Pokhara, Nepal; 5 International Medical University, Bukit Jalil, Kuala Lumpur, Malaysia

**Keywords:** Tobacco consumption, Epidemiology, Distribution, Socio-economic factors, Nepal

## Abstract

**Background:**

Nearly four-fifths of estimated 1.1 million smokers live in low or middle-income countries. We aimed to provide national estimates for Nepal on tobacco use prevalence, its distribution across demographic, socio-economic and spatial variables and correlates of tobacco use.

**Methods:**

A secondary data analysis of 2006 Nepal Demographic and Health Survey (DHS) was done. A representative sample of 9,036 households was selected by two-stage stratified, probability proportional to size (PPS) technique. We constructed three outcome variables 'tobacco smoke', 'tobacco chewer' and 'any tobacco use' based on four questions about tobacco use that were asked in DHS questionnaires. Socio-economic, demographic and spatial predictor variables were used. We computed overall prevalence for 'tobacco smoking', 'tobacco chewing' and 'any tobacco use' i.e. point estimates of prevalence rates, 95% confidence intervals (CIs) after adjustment for strata and clustering at primary sampling unit (PSU) level. For correlates of tobacco use, we used multivariate analysis to calculate adjusted odds ratios (AORs) and their 95% CIs. A p-value < 0.05 was considered as significant.

**Results:**

Total number of households, eligible women and men interviewed was 8707, 10793 and 4397 respectively. The overall prevalence for 'any tobacco use', 'tobacco smoking' and 'tobacco chewing' were 30.3% (95% CI 28.9, 31.7), 20.7% (95% CI 19.5, 22.0) and 14.6% (95% CI 13.5, 15.7) respectively. Prevalence among men was significantly higher than women for 'any tobacco use' (56.5% versus 19.6%), 'tobacco smoking' (32.8% versus 15.8%) and 'tobacco chewing' (38.0% versus 5.0%). By multivariate analysis, older adults, men, lesser educated and those with lower wealth quintiles were more likely to be using all forms of tobacco. Divorced, separated, and widowed were more likely to smoke (OR 1.49, 95% CI 1.14, 1.94) and chew tobacco (OR 1.36, 95% CI 0.97, 1.93) as compared to those who were currently married. Prevalence of 'tobacco chewing' was higher in eastern region (19.7%) and terai/plains (16.2%). 'Tobacco smoking' and 'any tobacco use' were higher in rural areas, mid-western and far western and mountainous areas.

**Conclusions:**

Prevalence of tobacco use is considerably high among Nepalese people. Demographic and socioeconomic determinants and spatial distribution should be considered while planning tobacco control interventions.

## Background

Globally consumption of tobacco is a major risk factor for mortality [1-3] with an estimated five million people killed every year [4,5]. Smoking is known to cause mortality due to cardiovascular diseases, cancer and respiratory conditions [6-8]. It has been estimated that four-fifths of the estimated 1.1 million smokers live in low-and middle-income countries (LMICs) [4]. In Nepal, proportional mortality from chronic non-communicable diseases (NCDs) is 42% [9]. Such high mortality may be as a result of high smoking rates in the population aged 18 years and above [10]. The reported prevalence rate of tobacco use from small scale surveys ranges from 20% to 72% among different population groups [11-15]. The only large scale study from Nepal is the Global Youth Tobacco Survey (GYTS). However, this survey was done only in the Central Development Region among secondary school students [16].

The main form of tobacco consumption in Nepal is cigarettes; though another type of smoking tobacco i.e. *'bidis' *(hand-rolled cigarettes that contain unprocessed tobacco) is common in neighbouring India [17]. In south and south-east Asian countries particularly in India, tobacco is also consumed in various smokeless forms like chewing and sniffing. For example, '*paan masala' *(a balanced mixture of betel leaf with lime, areca nut, clove, cardamom, mint, tobacco, essence and other ingredients ), '*betel quid' *(mixture of areca nut, slaked lime and flavouring ingredients which are wrapped in betel leaf), *'gutka' *(a mixture of crushed areca nut, tobacco, catechu, paraffin, lime and sweet or savory flavourings), '*mishri' *(tobacco containing teeth cleaning powder) and '*snuff' *(pulverised tobacco leaves to be insufflated or "snuffed" through the nose) are commonly used smokeless tobacco forms which are prevalent in Nepal also [18]. Tobacco chewing is not only known to cause oral cancers [19] but also coronary heart disease [20]. Reliable data on cancer incidence for Nepal are not available. However, a hospital based study has shown that tobacco related cancers accounted for nearly half of all cancers among males and a quarter among females [21]. It has been reported that tobacco use is not uniform and is more common among lower socio-economic groups [22,23]. However, there could be differences in tobacco use according to demographic and spatial factors.

Nationally representative prevalence estimates and distribution of tobacco use have not been reported from Nepal. Such information would be useful to design national level policies and specific interventions for tobacco control within the country. The reported small scale studies do not provide such critical information. In this report, we have used the data from Nepal Demographic Health Survey (DHS) 2006 to provide national estimates for prevalence of tobacco use, its distribution across demographic, socio-economic and spatial variables and correlates of tobacco use.

## Methods

We conducted a secondary data analysis of Nepal DHS 2006, which is the third in the worldwide Demographic and Health Surveys (DHS) program. The survey was conducted under the administrative supervision of the population division of the Ministry of Health and Population (MOHP) and implemented by New ERA, a local research organization (http://www.newera.com.np/). Nepal DHS was technically supported by ORC (Opinion Research Corporation) Macro International Inc., and financially supported by the United States Agency for International Development (USAID). The field work for data collection was carried out between February and August, 2006.

### Sampling and sample size

The sampling frame for Nepal DHS 2006 was provided by a list of census enumeration areas with population and household information provided by the Population Census conducted in 2001. Nepal is divided into 14 zones and in total has 75 districts. Each district is further divided into Village Development Committees (VDCs), and each VDC into wards. The primary sampling (PSU) unit was ward, sub ward, or group of wards in rural areas, and sub wards in urban areas. The final sample size was 9,036 households which were selected by two-stage stratified, probability proportional to size (PPS) sampling method. In the first stage, a total of 260 PSU i.e. 82 in urban areas and 178 in rural areas were selected by systematic sampling using the PPS technique. At the second stage, enumeration of households in the selected wards was done. In each ward an average of 30 households in urban areas and 36 households in rural areas were selected by systematic sampling. Within each selected household, all women aged 15 to 49 years and men aged 15 to 59 years were eligible to be respondents for the survey.

The survey collected information about demographic factors, socio-economic factors and health status from eligible members in the sample of households selected. Data were collected according to a standard protocol. The three core survey questionnaires i.e. the Household Questionnaire, the Woman's Questionnaire and the Man's Questionnaire were translated into three local languages (Nepali, Bhojpuri and Maithili) and all were field tested. The questionnaire was administered to all eligible members of the household by face-to-face interview by the trained interviewers. The household questionnaire was administered to the respondents who reported to be the head of the household. They were at first asked to list all the usual residents and visitors who stayed in the household the night before the interview. Information about tobacco use was asked from all eligible female and male members. Questions about tobacco use asked to all male and female members who participated in the Nepal DHS. Further details of sampling design, training of survey team, survey management and quality control measures are separately documented in the country reports published by ORC Macro International[24].

### Outcome variables

Information about tobacco use was obtained from the following four questions that were asked in both men's and women's questionnaires of DHS:

1) Do you currently smoke cigarettes? (Response as 'yes' or 'no')

2) In the last 24 hours, how many cigarettes did you smoke?

3) Do you currently smoke or use any other type of tobacco? (Response as 'yes' or 'no')

4) What (other) type of tobacco do you currently smoke or use? (The options given were pipe, chewing tobacco, snuff and others).

We created three dichotomous outcome variables from the information obtained from the above questions. For our analysis, each respondent was classified as 'tobacco smoker', if the response to the first question was 'yes' or response to question four was 'pipe'. The respondent was classified as 'tobacco chewer' if the response to question four was 'chewing tobacco'. We combined the variables about the participants who smoke and/or chew tobacco including other forms like snuff to construct a new variable 'any tobacco use' to assess the distribution of tobacco consumption in any form. If the response for questions one or three was 'yes', then the respondent was classified as 'any tobacco user'.

### Explanatory variables

The socioeconomic, demographic and spatial factors were defined at three levels i.e. individual, household and spatial. Factors at individual level were age, gender, marital status, ethnic group/caste, religion and educational attainment. Caste is the basis of social hierarchical structure followed in Hindu religion which is the predominant religion followed in Nepal similar to the caste system that is existing in neighbouring India. However categorisation of more than 100 castes is very complex. So we did not use caste system in our analysis. At household level, wealth index which was calculated based on material possessions was considered. The spatial variables considered were urban or rural type of residence, development region, ecological zone i.e. mountain, hill or plains/terai. For administrative purpose, Nepal is divided into five developmental regions i.e. Eastern, Central, Western, Mid-western and Far-western. We divided religion followed into three categories: Hindu, Buddhist and Others (Muslim, Kirant, Christian etc). Wealth index is a relative index of household wealth. It was calculated based on a standard set of household assets, dwelling characteristics and ownership of consumer items as observed by the interviewer. The individuals were ranked on the basis of their household score and divided into quintiles where first quintile is the poorest 20% of the households and fifth quintile is the wealthiest 20% of the households[25].

### Ethical considerations

The Independent Review Boards (IRB) of IPPS and ORC Macro international had independently reviewed the DHS protocols and data collection procedures and provided ethical approval.

### Statistical analysis

Statistical analysis was carried out using SPSS (Statistical Package for Social Sciences) version-17. Existing variables were re-coded to create new variables for logistic regression analysis. We used 'complex samples' option to carry out the analysis in SPSS for multistage sampling used in DHS. To test the fixed effects of explanatory variables on tobacco use logistic regression analyses was done. To adjust for cluster sampling (cluster as primary sampling unit used in DHS) a weighting factor 'sample weight' was used [26]. Univariate analysis was carried out to describe the sampled population according to demographic, socio-economic and spatial variables. We computed the overall prevalence of tobacco smoking, tobacco chewing and any tobacco use. We calculated the point estimates of prevalence rates, their robust 95% confidence intervals (CIs) after adjustment for strata and clustering at primary sampling unit (PSU) level. Binary logistic regression models were built to estimate the adjusted odds ratios (AOR) for association between tobacco consumption and explanatory variables. A p-value less than 0.05 was considered as significant.

## Results

The overall response rates for households, eligible women and eligible men were 99.6%, 98.4% and 96.0% respectively. The total number of households, eligible women and eligible men interviewed was 8707, 10793 and 4397 respectively. The descriptive statistics of the survey participants, the prevalence estimates and their 95% CIs for tobacco smoking (cigarettes), tobacco chewing and any form of tobacco use according to demographic, socio-economic and spatial variables are shown in table 1. The overall prevalence rates and their 95% CIs for any tobacco use, tobacco smoking and tobacco chewing were 30.3% (95% CI 28.9, 31.7), 20.7% (95% CI 19.5, 22.0) and 14.6% (95% CI 13.5, 15.7) respectively. The median number of cigarettes smoked was 5.0 (Q1=3 and Q3=10) (mean 7.1, standard deviation 5.94). Men were more likely to be using all forms of tobacco. Prevalence of any tobacco use among men (56.5%) was nearly three times that among women i.e. 19.6% (Table 1). Similarly prevalence smoking among men (32.8%) was double the prevalence among women (15.8%) while prevalence of tobacco chewing among men (38.0%) was nearly eight times higher than the prevalence of 5.0% among women (table 1).

**Table 1:** Descriptive statistics of background characteristics, prevalence and 95% confidence intervals of smoking, chewing and any tobacco use among survey participants included in the analysis

Characteristic	Participants (%)	Prevalence of Tobacco smoking	Prevalence of tobacco chewing	Prevalence of any tobacco use
Overall	15190	20.7 (19.5, 22.0)	14.6 (13.5, 15.7)	30.3 (28.9, 31.7)

**Age groups (in years)**				

14-19	3376 (22.2)	4.3 (3.5,5.4)	4.7 (3.7, 5.8)	7.7 (6.6, 9.0)

20-29	4989 (32.8)	12.0 (10.7, 13.4)	11.9 (10.8, 13.2)	20.5 (18.9, 22.2)

30-39	3485 (22.9)	27.5 (25.3, 29.8)	18.8 (17.0, 20.7)	39.6 (37.2, 42.1)

40-49	2785 (18.3)	41.4 (38.8, 44.0)	19.7 (17.5, 22.2)	53.3 (50.6, 56.0)

50-59	555 (3.7)	50.0 (44.8, 55.2)	45.5 (38.7, 52.5)	78.4 (73.5, 82.5)

**Sex**				

Male	4397 (28.9)	32.8 (30.6, 35.2)	38.0 (35.8, 40.3)	56.5 (54.1, 58.8)

Female	10793 (71.1)	15.8 (14.5, 17.1)	5.0 (4.3, 5.8)	19.6 (18.3, 21.0)

**Marital status**				

Currently married	11343 (74.7)	24.4 (22.9, 26.0)	16.9 (15.5, 18.3)	35.4 (33.7, 37.2)

Never married	3364 (22.1)	5.7 (4.7, 6.9)	6.3 (5.2, 7.5)	10.2 (8.9, 11.6)

Divorced/separated/widowed	483 (3.2)	39.4 (34.1, 45.1)	18.4 (14.5, 23.0)	50.3 (44.8, 55.9)

**Educational attainment**				

No education	6591 (43.4)	30.5 (28.7, 32.3)	13.4 (12.1, 14.8)	38.6 (36.7, 40.5)

Up to primary	3174 (20.9)	20.7 (18.8, 22.8)	21.1 (18.9, 23.4)	34.5 (32.0, 37.1)

Up to Secondary	4428 (29.2)	8.8 (7.4, 10.5)	12.6 (11.3, 14.0)	17.8 (15.9, 19.7)

Higher education	997 (6.6)	7.5 (5.6, 9.9)	10.4 (8.3, 13.0)	16.0 (13.3, 19.1)

**Religion**				

Hindu	13123 (86.4)	21.0 (19.7, 22.3)	14.4 (13.2, 15.6)	30.2 (28.7, 31.7)

Buddhist	1158 (7.6)	22.2 (18.8, 25.9)	14.7 (12.0, 17.9)	32.6 (28.6, 36.9)

Islam, Christian and others	909 (6.0)	15.7 (12.6, 19.4)	17.2 (13.8, 21.3)	28.5 (24.1, 33.3)

**Wealth Index**				

Poorest	3026 (19.9)	35.6 (33.0, 38.3)	16.4 (14.3, 18.7)	44.5 (41.7, 47.3)

Poorer	2805 (18.5)	23.7 (21.5, 26.0)	16.4 (14.4, 18.7)	33.6 (30.9, 36.3)

Middle	2693 (17.7)	20.4 (18.4, 22.6)	15.4 (13.4, 17.6)	30.7 (28.3, 33.2)

Richer	3252 (21.4)	15.3 (13.5, 17.2)	14.7 (13.0, 16.6)	26.5 (24.2, 28.9)

Richest	3414 (22.5)	11.9 (10.3, 13.7)	10.7 (9.1, 12.6)	19.4 (17.2, 21.9)

**Type of Residence**				

Urban	4249 (28.0)	15.3 (13.5, 17.3)	12.6 (10.9, 14.5)	23.9 (21.5, 26.5)

Rural	10941 (72.0)	21.8 (20.4, 23.3)	14.9 (13.7, 16.3)	31.5 (29.3, 33.2

**Development region**				

Eastern	3555 (23.4)	16.5 (14.4, 18.7)	19.7 (17.3, 22.4)	31.0 (28.0, 34.1)

Central	3902 (25.7)	22.5 (20.1, 25.1)	12.5 (10.8, 14.4)	29.8 (27.0, 32.8)

Western	2996 (19.7)	15.1 (13.1, 17.3)	15.6 (13.4, 18.1)	26.7 (23.9, 29.7)

Mid-western	2352 (15.5)	27.8 (23.5, 32.6)	10.1 (8.2, 12.3)	33.7 (29.4, 38.3)

Far-western	2385 (15.7)	25.2 (21.7, 29.1)	13.6 (9.5, 19.2)	32.4 (27.1, 38.1)

**Ecological zone**				

Mountain	2075 (13.7)	33.4 (28.2, 39.0)	11.5 (9.2, 14.4)	39.9 (34.8, 45.3)

Hill	5906 (38.9)	21.8 (19.8, 24.0)	13.1 (11.6, 14.7)	30.8 (28.5, 33.2)

Terai (plains)	7209 (47.5)	18.1 (16.5, 19.8)	16.2 (14.4, 18.1)	28.6 (26.4, 30.8)

### Distribution of tobacco consumption by spatial factors

The prevalence of all types of tobacco consumption varied significantly by spatial factors (tables 1). Prevalence rates for all forms of tobacco consumption were higher in rural areas as compared with urban areas. While prevalence of cigarette smoking was highest in mountainous areas (33.5%) and lowest in plains (18.1%); prevalence of tobacco chewing was highest in plains (16.2%) and lowest in the mountainous areas (11.5%). Prevalence of any tobacco use was highest in mountainous areas (39.9%). The regional pattern of tobacco consumption was also statistically significant. The Mid-western (33.7%) and Far-western regions (32.4%) had highest prevalence for any tobacco use. Eastern (16.5%) and western (15.1%) regions had lowest prevalence of smoking while mid-western (10.1%) and central (12.5%) regions had lower prevalence of tobacco chewing (table 1)

### Demographic and socioeconomic correlates of tobacco consumption by univariate analysis

Age was a very significant predictor of tobacco consumption in all forms. For every 10 years increase in age, the risk of smoking increased nearly two-folds (data not shown). For all forms of tobacco use, the risk of tobacco consumption increased very steeply after 40 years of age. Men were more likely to smoke (OR 2.61, 95% CI 2.27, 2.98) and chew tobacco (OR 11.63, 95% CI 9.66, 13.99) than women. Prevalence rates and risk for both tobacco smoking and chewing was significantly higher among divorced/separated/widowed, less educated, poorer segments, and rural areas as compared to currently married, more educated, wealthier segments and urban areas respectively. Such gradients in prevalence rates of tobacco use according to wealth index and education were less prominent for tobacco chewing than for tobacco smoking (table 1). Divorced, separated, widowed were more likely to smoke as compared to those who were currently married (OR 10.86, 95% CI 8.01, 14.72). Similarly divorced, separated, and widowed were more likely to chew tobacco (OR 3.37, 95% CI 2.38, 4.76) and use any form of tobacco (OR 8.96, 95% CI 6.84, 11.74). Individuals from households with poorest wealth quintiles were more likely to smoke (OR 4.09, 95% CI 3.34, 5.02) and chew tobacco (OR 1.64, 95% CI 1.28, 2.09) as compared to those from households with highest wealth quintiles. Individuals with no education were more likely to smoke (OR 5.45, 95% CI 3.96, 7.49) and chew tobacco (OR 1.33, 95% CI 1.01, 1.77) than those individuals with higher education. Interestingly, there were no significance differences for tobacco consumption according to religion.

### Socio-demographic, economic and spatial correlates of tobacco consumption by logistic regression analyses

Logistic regression analyses was done to test the associations between dependant variables of tobacco consumption and all the independent variables tested in univariate analysis. Though most of the variables remained significant for tobacco smoking, the effect size decreased considerably. The effect size for association of tobacco consumption decreased drastically for age, while that for gender increased drastically. Tobacco chewing, marital status and type of residence (urban/rural) were not significant after adjustment in multivariable model. The effect size for association of tobacco chewing with age and wealth quintiles decreased while that for gender and education increased. After adjustment, we also observed that individuals who were never married and those living in mountainous regions were less likely to chew tobacco. Religion was not associated with tobacco consumption even after adjustment for potential confounding variables (table 2).

**Table 2:** Correlates of tobacco smoking, chewing and any tobacco use by logistic regression analysis

Characteristic	Tobacco smoking AOR (95% CI)	Tobacco chewing AOR (95% CI)	Any tobacco use AOR (95% CI)
**Age groups**			

14-19	1	1	1

20-29	2.56 (1.84, 3.58) *	2.50 (1.83, 3.42) *	2.86 (2.23, 3.68) *

30-39	5.84 (4.18, 8.16) *	3.54 (2.48, 5.04) *	6.27 (4.81, 8.17) *

40-49	9.83 (6.95, 13.90) *	3.22 (2.27, 4.57) *	9.97 (7.53, 13.25) *

50-59	6.11 (4.03, 9.28) *	2.39 (1.52, 3.76) *	6.56 (4.44, 9.69) *

**Sex**			

Female	1	1	1

Male	5.01 (4.24, 5.91) *	17.77 (14.59, 21.69) *	14.68 (12.43, 17.33) *

**Marital status**			

Currently married	1	1	1

Never married	0.87 (0.63, 1.19) ×	0.500 (0.36, 0.69) *	0.62 (0.48, 0.80) *

Divorced/separated/widowed	1.49 (1.14, 1.94) †	1.36 (0.97, 1.93) ×	1.54 (1.19, 1.99) *

**Educational attainment**			

Higher education	1	1	1

Up to Secondary	1.73 (1.19, 2.49) *	1.81 (1.34, 2.45) *	1.93 (1.46, 2.54) *

Up to primary	3.34 (2.34, 4.77) *	2.83 (2.08, 3.84) *	4.19 (3.17, 5.55) *

No education	6.57 (4.59, 9.41) *	3.08 (2.21, 4.29) *	7.58 (5.61, 10.05) *

**Religion**			

Hindu	1	1	1

Buddhist	0.80 (0.62, 1.04) ×	1.04 (0.78, 1.39)×	0.93 (0.72, 1.20) ×

Islam, Christian and others	0.76 (0.57, 1.02) ×	1.08 (0.79, 1.46)×	0.89 (0.69, 1.17) ×

**Wealth Index**			

Richest	1	1	1

Richer	1.16 (0.92, 1.46) *	1.20 (0.92, 1.57) ×	1.33 (1.06, 1.67) *

Middle	1.56 (1.21, 2.00) *	1.40 (1.06, 1.86)†	1.67 (1.30, 2.14) *

Poorer	1.77 (1.37, 2.28) *	1.51 (1.14, 2.00) †	1.83 (1.42, 2.36) *

Poorest	2.74 (2.08, 3.61) *	1.71 (1.23, 2.37) †	2.92 (2.22, 3.84) *

**Type of residence**			

Urban	1	1	1

Rural	1.25 (1.01, 1.54) †	1.18, (0.93, 1.49)×	1.26 (1.03, 1.54) †

**Development region**			

Eastern	1	1	1

Central	1.46 (1.20, 1.78) *	0.45 (0.36, 0.58) *	0.81 (0.67, 0.97) †

Western	0.89 (0.70, 1.12) ×	0.73 (0.55, 0.96) *	0.75 (0.60, 0.94) †

Mid-western	1.65 (1.29, 2.11) *	0.34 (0.26, 0.46) *	0.89 (0.69, 1.14) ×

Far-western	1.45 (1.17, 1.79) *	0.51 (0.37, 0.69) *	0.84 (0.66, 1.06) ×

**Ecological zone**			

Terai (plains)	1	1	1

Hill	1.45 (1.23, 1.71) *	0.89 (0.73, 1.09) ×	1.57 (1.23, 2.05) *

Mountain	1.95 (1.52, 2.50 *	0.61 (0.43, 0.84) *	1.36 (1.17, 1.59) *

† p < 0.05, * p < 0.001, ×p > 0.05

The older individuals had higher odds of smoking and chewing tobacco as compared to the younger. The association between tobacco consumption and age had decreased after adjustment. The risk for smoking increased nearly twice up to the age of 40 years while risk for chewing tobacco did not show such gradient with age. After adjustment for other variables men were five times more likely to smoke, and 17.7 times more likely to chew tobacco than the women. Divorced, separated, and widowed were 1.5 times more likely to smoke as compared to those who were currently married. Similarly, divorced, separated, and widowed were 1.54 times more likely to use any form of tobacco. On the other hand never married/single were less likely to chew tobacco (OR 0.50, 95% CI 0.36, 0.69) or use any form of tobacco (OR 0.62, 95% CI 0.48, 0.80). Individuals who had no education were 6.57 times more likely to smoke and 3.1 times more likely to chew tobacco than those who had higher education. Individuals in the households with poorest quintiles were 2.74 times more likely to smoke and 1.71 times more likely to chew tobacco as compared to their counterparts from households with richest quintiles. People living in rural areas were 1.25 times more likely to smoke tobacco than their counterparts from urban areas. Among the developmental regions, tobacco smoking was more likely in mid-western (OR 1.65, 95% CI 1.29, 2.11) and far-western regions (OR 1.45, 95% CI 1.17, 1.79) than other regions of Nepal after adjustment. Such regional differentials were also statistically significant for tobacco chewing (table 2). Tobacco smoking was 1.95 times more likely among people in mountainous region than those in terai region while tobacco chewing was 0.65 times less likely in mountainous regions. To check for multicollinearity among spatial variables we calculated *tolerance and variation inflation factor (VIF). Tolerance was 0.998 and VIF was 1.002 *(Eigen value was 2.89 and condition index ranged from 5 to12).

## Discussion

This is the first scientific publication about tobacco consumption on a nationally representative sample of Nepal. Our report fills the gaps in the existing literature from small scale surveys about tobacco consumption in Nepal [11-13,15,16]. Our analysis has revealed that the distribution of tobacco consumption in Nepal varied not only across the population subgroups but spatially as well. Our results show that prevalence rates of tobacco consumption increase by age, tobacco consumption is predominantly a male habit especially among economically weaker and lesser educated population subgroups, thus highlighting the inequities in distribution of this important risk factor for chronic diseases.

### Limitations

The associations of tobacco consumption with socio-demographic, economic and spatial factors drawn from our statistical analyses lack a temporal relationship. This is due to the cross-sectional design used in Nepal DHS, thus limiting causal inference. The existing formal and informal social conventions about tobacco consumption in Nepal may have prevented participants of Nepal DHS from reporting their tobacco smoking and chewing habits. Nepal DHS, 2006 did not have any means to verify the self-reported tobacco use with biomarkers thus resulting in reporting bias. We used operational definitions for tobacco smoking, and tobacco chewing as we relied on the responses for four questions in DHS questionnaires. We could not classify tobacco smoking as 'ever smoker' 'current smoker', 'former smoker' and 'never smoker'. Global surveys on tobacco use i.e. Global Youth Tobacco Survey (GYTS), and Global Health Professional Student Survey (GHPSS), etc have used standard, validated questionnaires to obtain comparable data and classified the smoking status as categories mentioned above. Despite these limitations arising from secondary data and cross-sectional, observational study design, our results are important to guide future research on tobacco consumption and help health policy makers in planning tobacco control measures in Nepal.

### Prevalence estimates

Despite the lack of national level data, there are a few small scale surveys reporting about tobacco use in various population sub-groups of Nepal [11-16,27]. Our results are not comparable with the results of these studies. However, when compared to World Health Surveys (2002-04) done in 48 low-or middle-income countries (LMICs)[28], reported prevalence of current smoking in 2006 Nepal DHS is lower than prevalence reported from these 48 LMICs. The prevalence estimates from DHS in Sub Saharan countries [29]are similar to ours though the prevalence rates among individual countries varied from 8.0% to 27.3%. A National Sample Survey (NSS, 2000) which was carried out in 10 out of 75 districts has reported a prevalence of 'ever smoker' among males and females as 54.0% and 31.6% respectively [30]. This survey was done in 10 villages i.e. one village in each district covering a sample of 4400 individuals. The only other nationally representative survey data available is from Nepal DHS 2001[31] in which the questions about tobacco used were framed differently from the questionnaires for Nepal DHS, 2006 and NSS, 2000 [30,31]. In Nepal DHS 2001 and NSS, 2000 the questionnaires did not have questions about tobacco chewing and frequency of tobacco use. The first Nepal DHS, which was done in 1997, did not have any questions about tobacco use. The overall prevalence in Nepal DHS, 2006 is much lower than the reported prevalence of more than 50% for any form of tobacco use reported in Nepal DHS, 2001[31]. Such an over-estimate may have resulted from the method of questioning the participants. Though prevalence rates we report are much lower than that of NSS, 2000, the two rates cannot be compared since the definitions for smoking status, questionnaires and survey design were different. Moreover, DHS used 'current smoker' while NSS used 'ever smoker'. The prevalence rates reported among other small scale surveys are also much lower in other population sub groups i.e. college students, medical students, and school children [13,14,16]. A report based on India DHS 1998-99 has reported that the prevalence estimates of tobacco use may provide lower estimates due to under-reporting unlike the Nepal DHS 2001[22,31].

### Socio-demographic, economic and spatial variations

The increasing prevalence by age was similar to another report based on India DHS, 1998-99 [22]. This could not be attributed to increasing prevalence over time since we did not have comparable data. However, the prevalence rates according to age seem logical when compared to the results of our previous survey and other surveys from Nepal [11-13,15,16,27,30,32]. A plausible explanation for such a trend may due to cohort effect, i.e. smoking habit is less likely to be adopted in recent decades [22]. The striking gender differences observed in Nepal are not different from conservative societies of other Asian countries in where gender inequalities exist in other health indicators as well. The differentials in rates of tobacco use according to education and wealth quintile were significant even after adjustment. This may be due to a different socio-cultural milieu in which they live. The lesser educated people may not be aware of the health hazards of tobacco use and social acceptance of tobacco use may have predisposed them to tobacco smoking and chewing habits. There is very little or no implementation of information, education and communication activities (IEC) about health hazards in Nepal. This may be due to a lack of enforcement of comprehensive and strict tobacco control measures in Nepal though the government has ratified the Framework Convention for Tobacco Control (FCTC) in 2006 [33]. The spatial variation according developmental regions and ecological zones after controlling for other factors remained significant. A similar regional variation according to different states has been reported from neighbouring India [22,23]. We presume that higher prevalence of tobacco smoking in far and mid-western regions may be as a result of socio-economic and cultural factors which are distinct to these regions. We did not have data on these characteristics to be included in our analysis. People from terai areas (plain) and eastern regions both bordering India were more likely to be chewing tobacco, a practice prevalent in neighbouring India. In contrary to this people living in mountainous regions were more likely to be tobacco smokers. A previous survey from Jumla district and NSS, 2000 supports our results [30,34].

### Policy Implications

Our results support the need for specific approaches in tobacco control considering the variations in tobacco use in Nepal. Unlike the emphasis laid on preventing tobacco use among youth it may be important to address all age groups for effective tobacco control. Though rates of female tobacco use are lower, gender specific approaches should be considered, especially while transmitting messages on health promotion. Most importantly, higher rates among poor and the illiterate needs special attention without which they can be further impoverished due to money spend on buying tobacco products. It is important to start IEC activities to raise awareness about health hazards to tobacco use. This measure may complement the strategy of regulation and taxation implemented at the national level. Improving health of these poor cannot be achieved without effective tobacco control measures addressed towards them. There is an urgent need to *closely monitor of the progress in implementation in *certain regions and ecological zones which have higher proportions of tobacco smoking and chewing.

### Future research

To have a comparable data the definitions for tobacco use should be standardized. Regular population surveys with consistent definitions for tobacco use and robust survey designs should be carried out. Alternately a surveillance system to keep a watch on the trend of tobacco use and other important risk factors may be initiated. For example public policies may have an impact on distinct regional and socio-cultural factors. More insights into these factors and status of implementation of tobacco control measures in different regions may be useful for designing evidence based tobacco control interventions.

## Conclusion

Prevalence of tobacco use is still high, with a quarter of the Nepalese population consuming tobacco products. Considering the poor health indicators in Nepal, economic situation and an emerging threat of non-communicable disease burden, the Government of Nepal should seriously consider strict enforcement of tobacco control activities. While doing so the variations in tobacco use should be taken into account. Additionally, further research on epidemiology of tobacco use and monitoring of tobacco use epidemic would be useful in planning control strategies.

## Competing interests

The authors declare that they have no competing interests.

## Authors' contributions

CTS: Conceptualized the research, wrote the first draft of the manuscript for publication; NRR: Conceptualized the research, co-drafted the first draft of the manuscript for publication; HNHK: Helped conceptualizing the research, planned data analysis and revised earlier drafts of the manuscript; BS: Planned and conducted the data analysis, interpreted the results; JTA: Assisted in drafting the manuscript, commented draft versions of the manuscript for publication. All the authors and read and approved the final version of the manuscript to be submitted for publication in a scientific journal.

## References

[B1] World Health Organisation Tobacco or health: a global status report 1997 Geneva: World Health Organization

[B2] Ezzati M Lopez AD Estimates of global mortality attributable to smoking in 2000 Lancet 2003 362 847 852 10.1016/S0140-6736(03)14338-3 13678970

[B3] Lopez AD Mathers CD Ezzati M Jamison DT Murray CJ Global and regional burden of disease and risk factors, 2001: systematic analysis of population health data Lancet 2006 367 1747 1757 10.1016/S0140-6736(06)68770-9 16731270

[B4] Jha P Chaloupka FJ Moore J Gajalakshmi V Gupta PC Peck R Jamison DT, Breman J, Alleyne G, Claeson M, Evans D Disease Control Priorities in developing countries 2006 2 Oxford and New York: Oxford University Press 869 86 Tobacco addiction.

[B5] Jha P Avoidable global cancer deaths and total deaths from smoking Nat Rev Cancer 2009 9 655 664 10.1038/nrc2703 19693096

[B6] Ezzati M Lopez AD Regional, disease specific patterns of smoking-attributable mortality in 2000 Tob Control 2004 13 388 395 10.1136/tc.2003.005215 15564623 PMC1747946

[B7] Ezzati M Henley SJ Thun MJ Lopez AD Role of smoking in global and regional cardiovascular mortality Circulation 2005 112 489 497 10.1161/CIRCULATIONAHA.104.521708 16027251

[B8] Ezzati M Henley SJ Lopez AD Thun MJ Role of smoking in global and regional cancer epidemiology: current patterns and data needs Int J Cancer 2005 116 963 971 10.1002/ijc.21100 15880414

[B9] World Health Organization The impact of chronic diseases in Nepal WHO 2002

[B10] WHO South-East Asia Region WHO World Health Survey WHO Global InfoBase Version: 1 292beta 2001

[B11] Binu VS Subba SH Menezes RG Kumar G Ninan J Rana MS Smoking among Nepali youth--prevalence and predictors Asian Pac J Cancer Prev 2010 11 221 226 20593960

[B12] Niraula SR Tobacco use among women in Dharan, eastern Nepal J Health Popul Nutr 2004 22 68 74 15190814

[B13] Sreeramareddy CT Kishore P Paudel J Menezes RG Prevalence and correlates of tobacco use amongst junior collegiates in twin cities of western Nepal: a cross-sectional, questionnaire-based survey BMC Public Health 2008 8 97 10.1186/1471-2458-8-97 18366781 PMC2292712

[B14] Sreeramareddy CT Suri S Menezes RG Kumar HN Rahman M Islam MR Self-reported tobacco smoking practices among medical students and their perceptions towards training about tobacco smoking in medical curricula: A cross-sectional, questionnaire survey in Malaysia, India, Pakistan, Nepal, and Bangladesh Subst Abuse Treat Prev Policy 2010 5 29 10.1186/1747-597X-5-29 21080923 PMC2994841

[B15] Jha NP Upadhyaya MP Lakhey S Yadav BK Baral DD Gautam A Smoking and smokers in Sunsari, Nepal J Nep Med Assoc 1999 38 7 13

[B16] Pandey MRPR Challenges of tobacco use behavior in central development region of Nepal: Global Youth Tobacco Survey Collaborative Group Nepal GYTS Fact Sheet 2002

[B17] Shimkhada R Peabody JW Tobacco control in India Bull World Health Organ 2003 81 48 52 12640476 PMC2572308

[B18] Lee CH Ko AM Warnakulasuriya S Yin BL Zain RB Ibrahim SO Inter-country prevalences and practices of betel-quid use in south, south east and eastern asia regions and associated oral preneoplastic disorders: An international collaborative study by asian betel-quid consortium of south and east asia Int J Cancer 2010 129 1741 51 10.1002/ijc.25809 21128235

[B19] Critchley JA Unal B Health effects associated with smokeless tobacco: a systematic review Thorax 2003 58 435 443 10.1136/thorax.58.5.435 12728167 PMC1746661

[B20] Critchley JA Unal B Is smokeless tobacco a risk factor for coronary heart disease? A systematic review of epidemiological studies Eur J Cardiovasc Prev Rehabil 2004 11 101 112 10.1097/01.hjr.0000114971.39211.d7 15187813

[B21] Binu VS Chandrashekhar TS Subba SH Jacob S Kakria A Gangadharan P Cancer pattern in Western Nepal: a hospital based retrospective study Asian Pac J Cancer Prev 2007 8 183 186 17696728

[B22] Rani M Bonu S Jha P Nguyen SN Jamjoum L Tobacco use in India: prevalence and predictors of smoking and chewing in a national cross sectional household survey Tob Control 2003 12 e4 10.1136/tc.12.4.e4 14660785 PMC1747786

[B23] Subramanian SV Nandy S Kelly M Gordon D Davey SG Patterns and distribution of tobacco consumption in India: cross sectional multilevel evidence from the 1998-9 national family health survey BMJ 2004 328 801 806 10.1136/bmj.328.7443.801 15070637 PMC383376

[B24] Ministry of Health and Population (MOHP), New ERA, Macrointernational Nepal Demographic and Health Survey 2006 2006 Ministry of Health and Population, New ERA, and Macro International Inc

[B25] Howe LD Hargreaves JR Huttly SR Issues in the construction of wealth indices for the measurement of socio-economic position in low-income countries Emerg Themes Epidemiol 2008 5 3 10.1186/1742-7622-5-3 18234082 PMC2248177

[B26] Levy PS Lemeshow S Sampling of Populations 2011 New York, John Wiley & Sons

[B27] Subba SH Binu VS Menezes RG Ninan J Rana MS Tobacco chewing and associated factors among youth of Western Nepal: A cross-sectional study Indian J Community Med 2011 36 128 132 10.4103/0970-0218.84132 21976798 PMC3180938

[B28] Hosseinpoor AR Parker LA Tursan dE Chatterji S Social determinants of smoking in low- and middle-income countries: results from the World Health Survey PLoS One 2011 6 e20331 10.1371/journal.pone.0020331 21655299 PMC3105024

[B29] Pampel F Tobacco use in sub-Sahara Africa: estimates from the demographic health surveys Soc Sci Med 2008 66 1772 1783 10.1016/j.socscimed.2007.12.003 18249479 PMC2679748

[B30] World Bank.South-east Asia region Nepal smoking prevalence tobacco economy 2011 World Bank 2001.

[B31] Ministry of Health Government of Nepal, New Era and ORC Macro International Nepal Demographic and Health Survey, 2001 Calverton, Maryland, USA: Family Health Division, Ministry of Health; New ERA; and ORC Macro

[B32] Pandey MR Venkatramaiah SR Neupane RP Gautam A Epidemiological study of tobacco smoking behaviour among young people in a rural community of the hill region of Nepal with special reference to attitude and beliefs Community Med 1987 9 110 120 3497008

[B33] Ministry of Health and Population, Government of Nepal The National Anti-Tobacco Communication. Campaign Strategy For Nepal 2011

[B34] Pandey MR Neupane RP Gautam A Epidemiological study of tobacco smoking behaviour among adults in a rural community of the hill region of Nepal with special reference to attitude and beliefs Int J Epidemiol 1988 17 535 541 10.1093/ije/17.3.535 3209333

